# Beyond the Needle: Is Liquid Biopsy the Future of Veterinary Medicine?

**DOI:** 10.3390/ijms27188183

**Published:** 2026-09-14

**Authors:** Iga Horodyska, Patrycja Kasperska, Daria Będkowska, Sara Al-Ameri, Aleksandra Adam, Izabela Herman, Marta Miszczak, Joanna Bubak

**Affiliations:** 1 EZA Student Science Club, Department of Epizootiology and Clinic of Birds and Exotic Animals, Division of Infectious Diseases and Veterinary Administration, The Faculty of Veterinary Medicine in Wroclaw, Wrocław University of Environmental and Life Sciences, Grunwaldzki Sq. 45, 50-366 Wrocław, Poland; 120498@student.upwr.edu.pl (I.H.); 120488@student.upwr.edu.pl (P.K.); dariabedkowska2@gmail.com (D.B.); 118225@student.upwr.edu.pl (S.A.-A.); 121754@student.upwr.edu.pl (A.A.); 2 Vetspec Companion Animal Healthcare Centre, Odrzanska 9, 55-003 Czernica, Poland; izabela.herman@o2.pl; 3 Department of Epizootiology and Clinic of Birds and Exotic Animals, Division of Infectious Diseases and Veterinary Administration, The Faculty of Veterinary Medicine in Wroclaw, Wrocław University of Environmental and Life Sciences, Grunwaldzki Sq. 45, 50-366 Wrocław, Poland; marta.miszczak@upwr.edu.pl; 4 Department of Pathology, Division of Pathomorphology and Veterinary Forensics, The Faculty of Veterinary Medicine in Wrocław, Wrocław University of Environmental and Life Sciences, 31 Norwida St., 50-375 Wrocław, Poland; 5 Interwet, Small Animal Veterinary Clinic, Ślężna 136 St., 53-111 Wrocław, Poland

**Keywords:** circulating tumor DNA, ctDNA, TEPs, EVs, CTCs, liquid biopsy, minimal residual disease, MRD, multi-screening tests, tumor

## Abstract

The detection of circulating tumor DNA (ctDNA) and circulating tumor cells (CTCs) is a minimally invasive approach for diagnosing and monitoring cancer. These liquid biopsy-based strategies enable the identification of primary or metastatic tumors and provide valuable information on tumor biology and treatment response. A variety of techniques are employed to analyze components obtained through liquid biopsy. While CTCs are typically detected using cell-based methods, ctDNA is commonly analyzed using highly sensitive molecular approaches, including quantitative PCR (qPCR), digital PCR (dPCR), and next-generation sequencing (NGS). Extracellular vesicles (EVs), which carry tumor-derived nucleic acids, proteins, and other biomolecules, are increasingly investigated as potential biomarkers, while tumor-educated platelets (TEPs) can reflect tumor-associated molecular changes and may provide additional information for cancer detection and monitoring. Furthermore, emerging multi-cancer early detection (MCED) approaches aim to identify molecular signatures associated with multiple cancer types from a single blood sample, highlighting the broader diagnostic potential of liquid biopsy. Due to the high specificity of tumors and somatic mutations, ctDNA serves as a real-time biomarker for tracking cancer progression. The advantages of ctDNA diagnostics include its low invasiveness and its capacity to detect cancer at early stages. In addition to confirming the presence of a tumor, liquid biopsy approaches facilitate the assessment of malignancy and the evaluation of treatment response. In the field of human medicine, ctDNA plays a pivotal role in diagnostic procedures. It is utilized not only for screening tests that detect the presence of cancer but also for the development of targeted treatment protocols for specific patients. These protocols are informed by the detection of genomic alterations and are designed to monitor the response to therapy over the course of treatment. Similarly, CTC, EV, TEP, and MCED approaches are being investigated as complementary tools for cancer detection, molecular characterization, prognosis, and longitudinal disease monitoring. In the domain of veterinary medicine, ctDNA has been instrumental in the diagnosis of various neoplasms, including osteosarcomas, hemangiosarcomas, lymphomas, canine mammary tumors, and melanomas. Other liquid biopsy components, including CTCs and EVs, also show promise for the detection and characterization of tumors in companion animals, although their clinical application remains less developed than in human medicine. Another significant application is in the detection of minimal residual disease (MRD), where a small number of cancer cells remain undetectable by conventional tests, such as blood counts. This underscores the significance of advanced ctDNA analysis. However, challenges persist, including the low concentration of ctDNA in blood, the low ratio of mutated to normal DNA fragments, potential contamination, biological variability, and the need for standardized protocols. This review synthesizes the current knowledge on liquid biopsy, including ctDNA, CTCs, EVs, TEPs, and emerging MCED approaches, and the potential for transferring these technologies from human medicine to animal medicine. Continued research is necessary to enhance the sensitivity and specificity of detection, which could facilitate early cancer diagnosis in animals and improve survival through timely treatment.

## 1. Introduction

Early cancer detection is a critical factor in determining therapeutic success and long-term survival in both human and veterinary oncology [1,2]. In many cases of malignancy, clinical symptoms manifest only after substantial tumor progression, limiting the efficacy of therapeutic intervention and reducing the likelihood of curative treatment. Consequently, modern oncology has shifted its focus towards the identification of minimally invasive diagnostic strategies capable of detecting molecular alterations associated with cancer development prior to the manifestation of overt clinical symptoms. In this context, liquid biopsy has emerged as a particularly promising diagnostic approach. This is due to its ability to enable real-time analysis of tumor-derived biomarkers obtained from body fluids without the need for invasive tissue sampling [3,4].

The concept of liquid biopsy emerged from advancements in the field of human oncology, where researchers sought minimally invasive methods capable of detecting and monitoring cancer using biomarkers circulating in body fluids as an alternative to traditional tissue biopsies [3]. A substantial body of research in the field of human medicine has demonstrated the potential of circulating tumor cells (CTCs), circulating tumor DNA (ctDNA), and other tumor-derived components, including extracellular vesicles (EVs), tumor-educated platelets (TEPs) and circulating cell-free RNA (cfRNA), to offer clinically relevant insights into tumor progression, metastasis, and therapeutic response [4,5]. In the context of human medicine, liquid biopsy is currently employed for several purposes, including early cancer detection, molecular characterization of tumors, monitoring treatment efficacy, identification of resistance mutations, and assessment of MRD following therapy [6]. The success of these techniques in human oncology has led to increased interest in these methods in veterinary medicine, particularly due to the molecular and biological similarities between canine and feline cancers and human cancers. It is important to note that liquid biopsy approaches have already been investigated in veterinary patients, predominantly in dogs and, to a lesser extent, in cats. In the field of canine oncology, cfDNA/ctDNA, CTCs, and EVs have been explored as potential biomarkers in a range of malignancies, including lymphoma, hemangiosarcoma, osteosarcoma, histiocytic sarcoma, mammary carcinoma, and urothelial carcinoma [1,7]. These studies have investigated potential applications in cancer detection, molecular characterization, prognostication, and monitoring of treatment response. In the field of feline oncology, the available evidence remains more limited and has focused mainly on cfDNA as a potential diagnostic and prognostic biomarker in cats with various tumors [8].

Overall, these findings indicate that liquid biopsy is already being evaluated in spontaneous cancers of companion animals, although its development remains less advanced than in human oncology. While some liquid biopsy-based approaches have reached clinical evaluation and commercial availability in canine oncology, most veterinary applications still require further validation before they can be routinely incorporated into clinical practice [1,7,9]. This has resulted in the emergence of comparative oncology as a significant translational research field [1].

The aim of this article is to provide an overview of liquid biopsy as an evolving, minimally invasive approach to cancer diagnostics, with particular emphasis on its potential applications in veterinary oncology. Based on advances achieved in human oncology, the current state of knowledge is presented, and the available veterinary literature is reviewed to evaluate the potential clinical utility of liquid biopsy in animal patients. Particular attention is given to its role in early cancer detection, disease monitoring, treatment selection, and assessment of therapeutic response, highlighting technologies that have already been validated and implemented in clinical practice. Established clinical applications of liquid biopsy in human oncology are considered alongside veterinary approaches, which are at different stages of investigation and clinical validation and, in most cases, have not yet been routinely implemented in veterinary practice. By comparing current applications and identifying opportunities as well as challenges associated with their implementation in veterinary medicine, the article provides a translational perspective on the future of liquid biopsy in veterinary clinical practice. Although this review does not present original experimental data, it offers a critical synthesis of the available literature and emphasizes how advances achieved in human oncology may support the development and wider adoption of liquid biopsy for the diagnosis and management of cancer in companion animals.

## 2. Methods for Cancer Detection—How Does It Work in Human Oncology?

In the field of human medicine, population-based screening programs have been instrumental in enhancing early cancer detection and reducing cancer-related mortality. Examples of such strategies include mammography, colonoscopy, low-dose computed tomography (LDCT), and prostate-specific antigen (PSA) testing, although their effectiveness in reducing cancer-related mortality varies depending on the cancer type, screening modality, and available evidence [10,11,12,13]. Despite the effectiveness of established screening programs, there remains a need for more sensitive and minimally invasive diagnostic tools capable of detecting cancer at its earliest stages, which has driven rapid progress in molecular diagnostics. Recent advancements in molecular diagnostics, high-throughput sequencing, bioinformatics, and nanotechnology have profoundly transformed cancer detection strategies. These developments have expanded the role of liquid biopsy from an experimental research tool to an increasingly clinically relevant platform supporting early diagnosis, molecular profiling, therapeutic monitoring, and MRD detection [14].

### 2.1. Conventional Diagnostic Approaches

Conventional diagnostic methods remain the cornerstone of cancer diagnosis and typically include physical examination, laboratory testing, diagnostic imaging, cytology, histopathology, and tissue biopsy [15]. Imaging modalities such as radiography, ultrasonography, computed tomography (CT), magnetic resonance imaging (MRI), and positron emission tomography (PET) are routinely used for tumor detection, staging, and treatment planning [16,17,18]. Histopathological evaluation of tissue biopsy specimens remains the gold standard for definitive diagnosis and tumor classification, while cytology may provide rapid preliminary assessment in selected cases [19,20]. However, tissue biopsy is inherently invasive, may not be feasible in all patients, and reflects only a limited portion of a biologically heterogeneous tumor, highlighting the need for complementary minimally invasive diagnostic approaches such as liquid biopsy.

### 2.2. Molecular Diagnostic Methods in Oncology

Advances in molecular oncology have substantially expanded the ability to detect cancer-associated genetic, epigenetic, transcriptomic, and proteomic alterations, supporting precision oncology through early diagnosis, prognostic assessment, therapeutic selection, and treatment monitoring [21,22,23,24,25]. Currently used molecular techniques include polymerase chain reaction (PCR)-based assays, digital PCR (dPCR), next-generation sequencing (NGS), fluorescence in situ hybridization (FISH), immunohistochemistry (IHC), and proteomic analyses [22,26,27,28,29]. Among these, dPCR and NGS have become particularly important for liquid biopsy applications due to their high sensitivity for detecting circulating tumor-derived biomarkers, including ctDNA and ctRNA [22,26,27,30,31,32]. Nevertheless, despite their high analytical performance, many molecular methods continue to rely on tissue sampling or are limited in their ability to capture the spatial and temporal heterogeneity of cancer, emphasizing the clinical value of minimally invasive liquid biopsy approaches [21,22,25,33].

### 2.3. Spectroscopic Approaches in Cancer Detection

Spectroscopic techniques, including Raman spectroscopy, surface-enhanced Raman spectroscopy (SERS), and Fourier-transform infrared spectroscopy (FTIR), have emerged as promising complementary tools for cancer detection by enabling rapid, label-free analysis of biochemical alterations in tissues and biological fluids [34,35,36,37,38,39,40,41]. These methods have shown encouraging results in identifying cancer-associated molecular signatures and are increasingly being investigated in combination with liquid biopsy biomarkers. Recent advances in molecular profiling and computational analysis have further enabled the development of multi-cancer early detection (MCED) assays, which integrate multiple biomarker classes to improve cancer detection are discussed in Section 5.

## 3. Liquid Biopsy Technologies

Liquid biopsy has emerged as one of the most rapidly developing areas in modern oncology due to its ability to provide minimally invasive and dynamic molecular information regarding tumor biology [21,22,23,25,33,42]. Unlike conventional tissue biopsy, liquid biopsy involves analysis of tumor-associated biomarkers present in body fluids circulating in body fluids such as blood, urine, saliva, cerebrospinal fluid, and pleural effusions [21,22,25,33].

The most extensively investigated liquid biopsy biomarkers include ctDNA, CTCs, EVs, circulating tumor RNA (ctRNA), microRNAs, and TEPs [22,25,33,43]. These biomarkers may reflect tumor burden, molecular heterogeneity, metastatic potential, treatment response, and emergence of resistance-associated alterations [21,22,23,24,25,42].

Among these biomarkers, ctDNA has received particular attention due to its potential utility in early cancer detection, molecular profiling, MRD assessment, and longitudinal disease monitoring [22,24,27,44]. Importantly, ctDNA analysis may enable identification of clinically actionable mutations before radiological progression becomes detectable [24,27,32]. Similarly, CTCs provide valuable information regarding metastatic dissemination and tumor aggressiveness, whereas EVs may facilitate intercellular communication and carry diagnostically relevant nucleic acids, proteins, and lipids [45,46,47].

In human oncology, these applications have progressed from research settings to established clinical tools for selected indications. Liquid biopsy approaches are increasingly incorporated into clinical oncology workflows, particularly in lung, breast, colorectal, and prostate cancers, with US Food and Drug Administration (FDA)-approved assays used in selected clinical settings for companion diagnostics and therapeutic stratification [21,30,48]. These established applications provide an important clinical framework for the further development of liquid biopsy in other fields of oncology.

In veterinary oncology, by contrast, clinical implementation remains at an earlier stage. Liquid biopsy approaches have been investigated in several canine malignancies, including lymphoma, hemangiosarcoma, mammary tumors, oral malignant melanoma, and osteosarcoma. Studies in canine lymphoma have evaluated circulating cell-free DNA and extracellular vesicles as potential biomarkers for diagnosis, molecular characterization, and monitoring of treatment response [49,50]. In hemangiosarcoma, cfDNA-based approaches have been investigated for cancer detection and characterization, while cfDNA has also been explored as a potential biomarker in canine mammary tumors [51,52]. Liquid biopsy has further been evaluated for disease monitoring in canine oral malignant melanoma and for detection and longitudinal assessment of circulating tumor cells in dogs with osteosarcoma [8,53,54]. Although these studies provide evidence that liquid biopsy can be evaluated in naturally occurring canine cancers, the level of clinical evidence varies substantially among tumor types. Some applications have been investigated longitudinally in clinical patients, whereas others remain preliminary or primarily biomarker-focused. Most veterinary applications therefore remain investigational or under clinical validation and have not yet achieved the level of routine clinical implementation established in human oncology. Thus, although human oncology provides a mature clinical framework for the use of liquid biopsy, its translation to veterinary medicine remains an evolving field requiring further analytical and clinical validation.

Comparative oncology represents an especially important area linking human and veterinary liquid biopsy research. Companion animals naturally develop cancers that share biological and molecular similarities with human malignancies. Therefore, veterinary patients may provide valuable translational models that support the discovery and validation of biomarkers [55,56,57].

Despite substantial progress, liquid biopsy technologies continue to face important challenges, including low biomarker abundance in early-stage disease, preanalytical variability, limited assay standardization, and difficulties associated with distinguishing tumor-derived alterations from background biological noise [21,25,30,42]. Consequently, further validation and harmonization studies remain necessary before widespread routine clinical implementation becomes feasible across multiple cancer types [21,25,30].

### 3.1. Emerging Technologies Supporting Liquid Biopsy

Recent advances in nanotechnology, biosensor engineering, microfluidics, and artificial intelligence have contributed to the development of liquid biopsy platforms with improved analytical performance and biomarker detection capabilities [35,36,37,38,45,47,58,59]. However, many of these approaches remain at the proof-of-concept or investigational stage, and their clinical applicability, particularly in veterinary oncology, requires further validation.

Nanotechnology-based biosensors are increasingly investigated for ultrasensitive detection of circulating cancer biomarkers [35,36,37,38,45]. Nanomaterials such as gold nanoparticles, magnetic nanoparticles, graphene derivatives, carbon nanotubes, and quantum dots enhance signal amplification, biomarker capture efficiency, and multiplex detection capabilities [35,37]. Integration of nanotechnology with spectroscopic platforms, electrochemical biosensors, and microfluidic systems has enabled the development of highly sensitive diagnostic devices capable of detecting extremely low concentrations of ctDNA, exosomal biomarkers, and circulating proteins [35,36,37,38,45].

Microfluidic technologies additionally facilitate rapid isolation and enrichment of CTCs and EVs from small-volume biological samples [35,37]. Such systems may improve automation, reduce processing time, and support development of point-of-care diagnostic platforms applicable to both human and veterinary oncology [1,37].

Artificial intelligence (AI) and machine learning algorithms have emerged as important tools for interpretation of complex multidimensional molecular datasets generated by liquid biopsy assays [47,58,59]. AI-assisted analysis may improve cancer classification, biomarker selection, prediction of treatment response, and early disease detection through integration of genomic, proteomic, imaging, and clinical data [47,58,59].

Collectively, these emerging technologies have the potential to enhance the sensitivity, specificity, accessibility, and clinical utility of liquid biopsy-based cancer diagnostics [35,36,37,38,45,58]. Their integration into both human and veterinary oncology may ultimately support earlier detection of malignancies, potentially enabling diagnosis before the onset of clinically apparent symptoms [1,45,57]. A comparative framework of diagnostic development is presented in Table 1.

### 3.2. Serum Protein Tumour Markers and Molecular Liquid Biopsy

In addition to the increasingly investigated molecular approaches, liquid biopsy in veterinary oncology also includes the measurement of circulating tumour-associated proteins in serum or plasma. Classical serum tumour markers, such as carcinoembryonic antigen (CEA), cancer antigen 15-3 (CA15-3), and other tumour-associated proteins, differ fundamentally from the newer molecular liquid-biopsy analytes. Protein biomarkers are generally released or shed by tumour cells, or may reflect host responses to the presence of a tumour, whereas molecular liquid biopsy approaches aim to detect tumour-derived nucleic acids, extracellular vesicles or intact tumour cells. Thus, these approaches provide complementary rather than interchangeable information [1,7,9,61,62].

Several serum protein markers have been investigated in canine cancer. CEA, for example, has been detected in canine serum and was reported to be increased in some dogs with mammary tumours; however, its diagnostic performance was not sufficiently consistent to support its use as a general cancer marker [7,63]. Similarly, serum CA15-3 has been investigated in canine mammary tumours. One study found increased CA15-3 concentrations in dogs with malignant mammary tumours, particularly in animals with lymph-node or distant metastases, whereas CEA concentrations did not differ significantly between the examined groups [63]. More recently, a study evaluating CEA, CA15-3 and cfDNA in dogs with mammary tumours reported a positive association between these biomarkers and cytological and histopathological tumour characteristics, with CA15-3 showing better diagnostic performance than CEA in that study [64]. These findings illustrate the potential value of serum protein markers, particularly for specific tumour types or disease monitoring, but also highlight the need for tumour-specific validation [1,7,61,64].

An important limitation of conventional circulating protein markers is their lack of tumour specificity. Many proteins can be detected in the circulation of healthy animals and may increase in response to inflammation, tissue injury or other non-neoplastic conditions. Consequently, elevated serum concentrations do not necessarily indicate the presence of cancer [1,7]. Reviews of canine cancer biomarkers have therefore concluded that no currently investigated circulating protein biomarker fulfills all the requirements of an ideal tumour marker, particularly with respect to sensitivity and specificity [1,7]. Protein biomarkers may nevertheless have clinical value in selected tumour types, especially when used together with clinical examination, imaging, cytology or histopathology, and when serial measurements are interpreted in the context of an already established diagnosis [1,7,9,61,62].

### 3.3. Alternative Biological Fluids in Veterinary Liquid Biopsy

Although blood is currently the most extensively investigated matrix for liquid biopsy, other biological fluids may also provide clinically relevant tumour-associated information. Liquid biopsy can encompass the analysis of urine, saliva, cerebrospinal fluid (CSF), serous effusions and other locally collected fluids for the detection of tumour-associated nucleic acids, proteins, metabolites, extracellular vesicles and, in selected settings, neoplastic cells [1,9,61]. The diagnostic value of these matrices is likely to depend strongly on tumour location and biological characteristics, because fluids that are anatomically close to a neoplasm may contain a higher concentration of tumour-derived material than peripheral blood [1,61]. However, the evidence supporting these approaches in veterinary medicine remains considerably less extensive than that available for blood-based assays, and most applications remain investigational rather than clinically established [1,9,61].

Urine represents one of the most promising alternative matrices because it can be collected non-invasively and repeatedly [1,9,61]. In canine oncology, urinary biomarkers have been investigated, particularly in urothelial carcinoma. Urinary extracellular vesicles from dogs with urothelial carcinoma showed increased levels of miR-182, miR-221 and miR-222 compared with healthy dogs and dogs with urinary tract infections, identifying these miRNAs as potential urinary biomarkers [65]. In another pilot study, increased concentrations of the urinary exosomal nuclear matrix protein NMP-22 were observed in dogs with urothelial carcinoma; however, the study included only six dogs with confirmed urothelial carcinoma, and the authors emphasized the need for larger studies [66]. Urinary proteomic profiling has also been investigated for canine transitional cell carcinoma. In a pilot study involving 12 dogs, 379 urinary proteins were identified, 96 of which were unique to the transitional cell carcinoma group, and a multiplex biomarker model predicted disease status with 90% accuracy [67]. More recently, Raman spectroscopy of urine was used to generate multimolecular spectral fingerprints capable of discriminating dogs with cancer from cancer-free dogs and, to some extent, distinguishing among tumour types [68]. Although the reported performance was promising, these findings should be interpreted as evidence of feasibility rather than as validation of a general-purpose clinical screening test. Larger prospective studies, external validation and assessment in clinically representative populations are still required [9,68].

Saliva may be particularly informative for tumours of the oral cavity because of its direct contact with neoplastic tissue [69,70,71]. Several studies in dogs have therefore investigated salivary proteomic and metabolomic profiles in oral neoplasia. Salivary proteomic analyses have identified candidate proteins associated with canine oral melanoma and oral squamous cell carcinoma, including PTPN5 and p53, while earlier MALDI-TOF and LC-MS/MS analyses identified additional candidate proteins and peptide fingerprints that differed among oral melanoma, oral squamous cell carcinoma, benign oral tumours and control groups [69,70]. More recent work has demonstrated differences in salivary metabolites between dogs with oral melanoma, oral squamous cell carcinoma, benign oral tumours and healthy controls, with candidate metabolites identified for both malignant tumour types [71]. Saliva has also been investigated for monitoring the response to treatment in canine oral melanoma, with differences in the salivary ubiquitin D profile observed during treatment and in relation to survival [72]. These studies support the concept that saliva may contain tumour-associated molecular signatures and may be useful as a readily accessible, non-invasive source of biomarkers, although the identified candidates remain investigational and require independent clinical validation before routine diagnostic use [69,70,71,72].

Cerebrospinal fluid may offer a different advantage because it is anatomically closer to tumours of the central nervous system (CNS) than peripheral blood [9,61]. In dogs with CNS neoplasia, increased concentrations of vascular endothelial growth factor (VEGF) have been detected in CSF. However, VEGF was also detectable in a substantial proportion of dogs with inflammatory CNS disease, and VEGF concentrations did not significantly distinguish the neoplastic from the inflammatory group, illustrating the difficulty of achieving tumour specificity [73]. More recent work has demonstrated alterations in several CSF biomarkers in dogs with brain tumours: tau and neuron-specific enolase (NSE) were increased, whereas amyloid-β was decreased compared with controls. Neurofilament light chain (NfL), in contrast, was particularly elevated in dogs with meningoencephalitis of unknown origin (MUO), highlighting that these biomarkers may reflect neurological injury or disease processes rather than being specific markers of neoplasia [74]. Thus, CSF may provide useful molecular information in selected patients with suspected CNS neoplasia; nevertheless, its invasive collection and the potential risks associated with CSF sampling, particularly in patients with intracranial masses or increased intracranial pressure, limit its applicability as a general screening matrix [9,61,74].

Serous effusions and other locally accumulated fluids may also be regarded as valuable liquid-biopsy matrices, particularly when neoplastic disease involves body cavities [9,61]. Pleural, peritoneal and other effusions can contain tumour cells that may be investigated by cytology, immunocytochemistry, cell-block immunohistochemistry, flow cytometry or molecular approaches [75,76]. In canine patients, neoplastic cells have been successfully characterized in body-cavity effusions using cell-block immunohistochemistry; for example, epithelial cells expressing progesterone receptor were identified in ascitic and pleural fluid from dogs with ovarian papillary adenocarcinoma [75]. Flow-cytometric approaches have also been investigated for the identification and characterization of non-haematopoietic cells in canine effusions. In a study of 36 canine effusions, flow cytometry using cytokeratin, vimentin and desmin provided results broadly concordant with immunohistochemistry, although discrepancies occurred, particularly for vimentin expression [76]. In addition, protein composition may provide ancillary information: total protein concentrations were higher in neoplastic than in non-neoplastic canine pleural and peritoneal effusions, although the considerable overlap between groups limited the diagnostic utility of this parameter [77]. These findings suggest that effusions may be particularly useful as a source of tumour cells or tumour-associated biomarkers when an effusion is already present, rather than as a general-purpose screening sample [75,76,77].

Importantly, the term “circulating tumour cells” should be used cautiously when discussing these alternative matrices. CTCs, by definition, refer to tumour cells detected in the circulation, whereas tumour cells recovered from an effusion or another locally collected fluid are more appropriately described as neoplastic or tumour cells unless their biological status and route of dissemination justify the term [61]. In veterinary medicine, even blood-based CTC analysis remains at an early stage of development. For example, a recent pilot study demonstrated the feasibility of detecting CTCs in the blood of dogs with oral malignant melanoma using size-based isolation followed by immunocytochemistry; intact cells were detected in 9 of 10 dogs, but the authors emphasized the preliminary nature of the findings and the need for larger studies [78]. Consequently, for alternative fluids, the strongest current veterinary evidence concerns the detection of tumour-associated molecular biomarkers and neoplastic cells rather than the routine enumeration of CTCs [9,61,78].

Taken together, these observations suggest that the future of veterinary liquid biopsy may extend beyond blood-based testing toward a more site-specific strategy in which the choice of biological fluid is guided by tumour location and biology [1,9,61]. Urine may be particularly relevant for urinary tract neoplasia, saliva for oral tumours, CSF for selected CNS disorders, and pleural or peritoneal effusions when neoplastic involvement of body cavities is suspected [9,61]. Such approaches could complement, rather than replace, conventional tissue biopsy and cytological or histopathological examination [9,61]. At present, however, most non-blood liquid-biopsy applications in veterinary oncology should be considered promising research tools rather than established clinical diagnostic tests [1,9,61].

## 4. Clinical Applications of Liquid Biopsy Biomarkers: Advantages and Drawbacks

Liquid biopsy represents a promising minimally invasive approach in veterinary oncology, with potential applications in cancer detection, disease characterization, longitudinal monitoring, and assessment of treatment response and minimal residual disease (MRD) [1,5]. However, the extent of clinical validation differs substantially among liquid biopsy analytes and between human and veterinary oncology. In veterinary patients, the most substantial species-specific evidence currently concerns ctDNA/cfDNA, for which canine studies have demonstrated the feasibility of detecting tumor-associated genomic alterations and have provided preliminary evidence for diagnostic applications, treatment-response monitoring, and MRD assessment in selected malignancies [1,5]. In particular, ctDNA has been detected in dogs with histiocytic sarcoma, oral malignant melanoma, and multicentric lymphoma. In a study by Prouteau et al., ctDNA was detected in 21/23 dogs with histiocytic sarcoma, 2/8 dogs with oral melanoma, and 12/13 dogs with lymphoma. Furthermore, plasma analysis of lymphoma-specific antigen receptor rearrangements in four dogs showed concordance between minimal residual disease detection, clinical evaluation, and treatment response, supporting the potential role of ctDNA in disease monitoring [79]. Nevertheless, these applications remain dependent on tumor type, assay design, and analytical sensitivity, and should not yet be regarded as universally validated diagnostic or monitoring tools across canine and feline cancers.

Evidence for CTCs and EVs in veterinary patients is also emerging, but is generally based on smaller, disease-specific studies. CTCs have been investigated in dogs with mammary carcinoma and osteosarcoma, with studies demonstrating the feasibility of detecting circulating malignant cells in peripheral blood and suggesting their potential relevance to metastatic disease [80,81]. However, standardized methods for CTC enrichment, identification, and enumeration have not yet been established across veterinary species or tumor types. EV-based assays have likewise demonstrated species-specific potential in canine oncology. For example, Garnica et al. evaluated serum small extracellular vesicles in dogs with multicentric lymphoma and found that EV concentration at diagnosis was associated with chemotherapy response. These findings provide species-specific proof of concept for EV-based liquid biopsy, but the clinical utility of EV-based assays remains investigational and requires validation in larger, independent cohorts [49].

In contrast, TEPs represent a substantially less mature area in veterinary oncology. Their diagnostic and prognostic potential has been demonstrated primarily in human cancer studies. For example, Best et al. demonstrated that RNA sequencing of tumor-educated platelets could distinguish cancer patients from healthy individuals with high accuracy and could provide information regarding the anatomical location and molecular characteristics of tumors [82]. Subsequent reviews have further highlighted the potential of TEPs as a liquid biopsy source while emphasizing the need for further clinical translation and standardization [83]. At present, evidence supporting the clinical application of TEP-based liquid biopsy in veterinary patients is considerably more limited than that available for ctDNA, CTCs, or EVs. Therefore, proposed applications of TEPs in veterinary oncology should currently be regarded largely as extrapolations from human oncology and as an area requiring species-specific validation.

Importantly, these biomarkers should not be considered interchangeable. Their biological signals differ fundamentally: ctDNA reflects tumor-derived genetic and epigenetic material; CTCs represent intact circulating malignant cells and may retain phenotypic and functional characteristics of the primary or metastatic tumor; EVs carry diverse molecular cargo, including nucleic acids, proteins, and lipids, and may reflect tumor–host communication; whereas TEPs reflect tumor-induced systemic alterations in circulating platelets, particularly changes in their RNA and protein composition [84,85,86]. Consequently, the diagnostic, prognostic, and monitoring value of each analyte is likely to depend not only on the tumor type and clinical question but also on the degree of species-specific analytical and clinical validation. Thus, while liquid biopsy is an emerging component of veterinary precision oncology, most applications remain investigational, with ctDNA currently providing some of the strongest clinical evidence and CTC-, EV-, and particularly TEP-based approaches requiring further prospective validation.

### 4.1. ctDNA

Among the available liquid biopsy biomarkers, ctDNA is one of the most extensively investigated and clinically developed approaches in human oncology, with established applications in selected settings, particularly for molecular characterization, treatment selection, and longitudinal disease monitoring [24,44,87]. In veterinary oncology, ctDNA has also been increasingly investigated, including for longitudinal disease monitoring; however, its clinical implementation remains substantially more limited, and the available evidence is largely derived from disease-specific clinical studies and analytical or clinical validation cohorts rather than routine use in veterinary practice [24,44,87]. A key advantage of liquid biopsy is the ability to obtain repeated samples with minimal invasiveness, enabling longitudinal monitoring of tumor dynamics and treatment response [46,88]. Changes in ctDNA levels may reflect treatment response, disease progression, or recurrence and may complement conventional imaging modalities during patient follow-up [22,88]. In addition, ctDNA analysis enables detection of tumor-specific genetic and epigenetic alterations, including somatic mutations and DNA methylation changes, which may provide clinically relevant prognostic information and support therapeutic stratification [24,87].

The use of ctDNA as a dynamic biomarker can be employed in treatment monitoring. Decreasing ctDNA levels are generally associated with a favorable therapeutic response, while increasing levels may indicate disease progression [88,89]. However, reduced assay sensitivity during treatment may also be considered a significant challenge [26,90]. Persistently elevated ctDNA levels despite therapy may be associated with an increased risk of recurrence and poor prognosis [88,91]. In patients undergoing surgical tumor resection, preoperative ctDNA levels have been correlated with recurrence rates and overall survival (OS), both of which are reduced in patients with high ctDNA concentrations [81]. Moreover, ctDNA integrity and methylation patterns have been associated with prognosis in various cancer types and may provide additional value in predicting disease outcome and guiding therapeutic decisions [92]. It is important to distinguish these ctDNA-specific applications from studies measuring total circulating cell-free DNA (cfDNA). For example, the study conducted in patients with head and neck cancer undergoing radiochemotherapy evaluated circulating cfDNA rather than tumor-specific ctDNA. Increased cfDNA levels were associated with concurrent infections and inflammatory processes, indicating that cfDNA may provide complementary information on treatment-related systemic effects; however, these findings should not be interpreted as evidence that ctDNA levels can distinguish local recurrence from infection or inflammation [93].

Despite these advantages, ctDNA analysis is associated with several important limitations. Pre-analytical variables, including blood collection, storage conditions, and processing time, may significantly influence assay performance. From a clinical workflow perspective, the volume of peripheral blood required for liquid biopsy analysis depends on the specific assay, collection tube, analytical platform, and clinical application. In veterinary patients, sample volume may also need to be adjusted according to body size and practical considerations. Regardless of the required volume, plasma separation should preferably be performed as soon as possible after blood collection to minimize leukocyte lysis and contamination with non-tumor cell-free DNA [30,94]. In addition, the anatomical site of blood collection may also influence the detection of tumour-derived material in liquid biopsy samples. In dogs with cancer, blood collected from the jugular vein has been shown to contain a higher tumour fraction than blood collected from peripheral veins, such as the cephalic or saphenous veins. A shorter circulation distance between the tumour and the blood collection site was also associated with a higher tumour fraction, suggesting that the anatomical sampling site should be considered as a potential pre-analytical factor in liquid-biopsy studies [95,96,97]. Similar effects of sampling site on the abundance and detectability of circulating tumor-derived material have also been investigated in human oncology [97].

Furthermore, ctDNA typically represents only a minor fraction of total circulating cell-free DNA (often below 1% in early-stage disease, although reported fractions range from approximately 0.01% to over 90% depending on tumor type and burden), requiring highly sensitive analytical platforms for reliable detection [43,44,98]. A major biological limitation is reduced ctDNA detectability in tumors with low burden, low proliferative activity, or limited vascularization. Although ctDNA detection generally decreases with decreasing tumor size, this relationship is highly context-dependent and varies according to tumor type, anatomical location, vascularity, biological aggressiveness, and disease stage. Studies conducted in specific cancer populations have reported markedly reduced detectability in lesions below 1 cm, while lesions in the 1–3 cm range have been associated with more favorable detection in some settings. These size ranges should therefore be considered context-specific rather than universal thresholds for ctDNA detection across malignancies [44,88].

Interpretation of ctDNA results may also be affected by non-neoplastic processes such as inflammation, tissue injury, or immune-mediated disease. These conditions can increase the concentration of total circulating cfDNA and may therefore alter the relative fraction of tumor-derived ctDNA, potentially affecting the sensitivity and specificity of ctDNA-based analyses [43]. In addition, theoretical models suggest that even highly accurate assays may have limited screening performance in low-prevalence settings. For example, an assay with 95% specificity and 100% sensitivity may still yield a positive predictive value of approximately 5%, resulting in a substantial proportion of false-positive findings and potential overdiagnosis [99,100].

In human oncology, ctDNA-based assays are therefore considered more suitable for disease monitoring, MRD detection, and prognostic assessment than for primary cancer screening. In human patients, circulating cfDNA concentrations may be influenced by various physiological and environmental factors, including smoking, pregnancy, cardiovascular disease, inflammation, physical activity, and diurnal rhythmic variation [30]. Importantly, these factors may affect the overall cfDNA pool and should not be interpreted as direct increases in tumor-derived ctDNA. Similar effects on circulating cfDNA concentrations have been reported in dogs, where conditions such as immune-mediated hemolytic anemia, trauma, and inflammatory diseases may contribute to increased cfDNA levels [79]. Thus, elevated cfDNA concentrations in dogs with non-neoplastic conditions should be distinguished from tumor-derived ctDNA signals when interpreting liquid biopsy results.

From an economic perspective, multi-cancer ctDNA screening approaches have been estimated at approximately £500 per test, which highlights both the potential clinical utility and cost considerations associated with broader implementation [101]. In veterinary medicine, the financial implications of introducing such diagnostic approaches may be particularly relevant, as the costs of diagnostic investigations and subsequent treatment are typically borne directly by the animal. Consequently, access to advanced diagnostic testing may be influenced not only by its clinical indication and expected diagnostic benefit, but also by the owner’s financial capacity and willingness to incur additional veterinary expenditure. This economic dimension may therefore represent an important consideration when evaluating the feasibility and wider adoption of ctDNA-based screening strategies in veterinary practice.

### 4.2. CTCs

CTCs are intact malignant cells released from primary or metastatic tumors into the bloodstream. Unlike ctDNA, CTCs provide direct cellular and phenotypic information, including morphology, protein expression, and cellular viability, thereby enabling detailed biological characterization and, in selected settings, downstream molecular analyses at the single-cell level. However, CTC analysis is technically challenging due to their extreme rarity, particularly in early-stage disease. Detection typically requires enrichment strategies based on physical properties, such as size or density, or biological properties, such as the expression of tumor-associated surface markers [102,103]. Importantly, reliance on epithelial markers may fail to detect tumor cells undergoing epithelial-to-mesenchymal transition, contributing to false-negative results [102,103]. CTCs also exhibit substantial biological heterogeneity, as circulating tumor cell populations may differ in epithelial and mesenchymal phenotypes, gene expression profiles, and functional or metastatic potential [104,105]. This variability limits standardization and reproducibility across analytical platforms [102].

In veterinary oncology, CTC-based approaches remain largely experimental, with limited validation studies and a lack of standardized protocols. Nevertheless, primary studies in dogs have demonstrated the feasibility of CTC detection and provided preliminary evidence of prognostic and monitoring value in selected malignancies. In canine metastatic mammary carcinoma, CTCs were detected using the CellSearch platform in 44.4% of evaluable blood samples, and CTC presence was significantly associated with poorer outcome [106]. In canine osteosarcoma, flow-cytometric enumeration of CTCs demonstrated that increased CTC frequency could precede radiographic detection of metastases and was associated with shorter survival [53,54]. More recently, CTCs were detected in 9 of 10 dogs with oral malignant melanoma using size-based isolation followed by immunocytochemistry, with preliminary evidence of an association between CTC count and lymph node metastasis [78]. These findings support the potential utility of CTCs for prognosis assessment and evaluation of metastatic potential in selected canine malignancies; however, the available evidence remains limited, disease-specific, and method-dependent, and further prospective studies are required before CTC-based assays can be incorporated into routine veterinary oncology practice.

### 4.3. EVs

EVs, including exosomes and microvesicles, are membrane-bound particles released by cells and present in various biological fluids, including blood, saliva, and urine [107,108]. EVs carry diverse molecular cargo, including proteins, lipids, DNA, mRNA, and microRNAs, reflecting characteristics of their cells of origin [107,108]. Encapsulation of nucleic acids and other biomolecules within the lipid bilayer of EVs can protect their cargo from extracellular enzymatic degradation, contributing to its stability [107,108]. EVs play an important role in intercellular communication within the tumor microenvironment and are involved in processes such as angiogenesis, immune modulation, and metastatic niche formation [108,109]. These properties support their potential as diagnostic and prognostic biomarkers. However, EV-based diagnostics face major methodological challenges. Isolation and characterization methods are not yet fully standardized, and different protocols can yield highly variable results in terms of EV yield, composition, and purity [107,108]. Additionally, distinguishing tumor-derived EVs from those originating from normal or inflammatory cells remains a significant limitation [107,108]. EV populations are highly heterogeneous in size, cellular origin, and molecular cargo, complicating data interpretation and biomarker validation [107,108]. In veterinary oncology, EV research is still at an early stage, and robust clinical validation is lacking [107,108].

### 4.4. TEPs

TEPs are an emerging class of liquid biopsy biomarkers, with most current evidence derived from human oncology and potential applications in veterinary medicine. Platelets interact with tumor cells through uptake and transfer of tumor-derived molecules, including RNA, proteins, and EVs, resulting in a tumor-associated reprogramming of their molecular content that reflects disease presence and progression [86,110,111].

In human medicine, TEPs are investigated as minimally invasive biomarkers for early cancer detection, tumor classification, monitoring of treatment response, and detection of disease recurrence [86,112,113]. Unlike CTCs, platelets are highly abundant in peripheral blood and can be readily isolated using standard procedures. Their RNA is relatively stable due to protection within the platelet cytoplasm and vesicular compartments, making TEPs a potentially robust source for transcriptomic profiling [86,111,114]. Importantly, platelets can acquire tumor-associated molecular and functional alterations through interactions with tumor cells and the tumor microenvironment, providing a distinct source of cancer-related information that may complement cell-free and cell-based liquid biopsy analytes. Several studies have reported promising diagnostic performance of TEP-derived RNA signatures across different malignancies, including lung, breast, colorectal, and pancreatic cancers, although most available data derive from early-phase or proof-of-concept studies [86,112,114].

In veterinary medicine, research on TEPs remains limited, and their application is currently largely exploratory. Naturally occurring cancers in dogs share many biological and clinical similarities with human malignancies, supporting their potential use as translational models for biomarker development. Preliminary studies in canine oncology suggest that platelet-related molecular alterations may have diagnostic and prognostic potential in naturally occurring cancers, including both solid and hematological malignancies; however, evidence specifically validating TEP-derived RNA signatures in veterinary patients remains scarce and requires further investigation [110,112]. The minimally invasive nature of liquid biopsy is particularly advantageous in veterinary practice, where repeated tissue sampling may be challenging or associated with increased procedural risk.

Despite their promise, several limitations currently hinder the routine clinical implementation of TEP-based diagnostics. A major challenge is the lack of standardized protocols for platelet isolation, RNA sequencing, and downstream bioinformatic analysis, which may contribute to variability and limited reproducibility between studies [112,113,115]. In addition, platelet molecular profiles can be influenced by non-malignant conditions, including systemic inflammation and thrombosis, which may reduce diagnostic specificity [110,116]. Therefore, further large-scale, multicenter validation studies are required before TEP-based assays can be reliably integrated into routine clinical practice [113,115,117].

Overall, TEPs represent a promising component of liquid biopsy approaches with substantial research interest and potential clinical applications in human oncology. In veterinary oncology, their potential remains largely exploratory, and their clinical utility has yet to be established. Further methodological standardization and clinical validation are therefore required before TEP-based assays can be considered for routine veterinary use [66,112,115].

### 4.5. Comparative Clinical Utility and Future Perspectives

It is evident that ctDNA, CTCs, EVs, and TEPs represent complementary liquid biopsy biomarkers, which capture different aspects of tumor biology. It has been established that ctDNA primarily reflects tumor-derived genetic and epigenetic alterations. In addition, CTCs provide intact cellular phenotypes with metastatic potential, EVs convey intercellular communication signals within the tumor microenvironment, and TEPs reflect systemic tumor-induced changes in circulating platelet RNA and protein profiles. Consequently, each analyte provides partially non-overlapping biological information, supporting their use as complementary rather than competing diagnostic tools [22,24,43,86,87,109,111,118].

Among these, ctDNA is currently the most clinically advanced biomarker and has been increasingly investigated for longitudinal monitoring of tumor burden and therapeutic response. CTCs, EVs, and TEPs remain at earlier stages of clinical translation, although experimental and early clinical studies suggest that they may provide complementary diagnostic and prognostic information. In canine cancer patients, analysis of EV-associated CDC6 mRNA has shown improved detectability compared with total plasma analysis, although the difference in Ct values did not reach statistical significance [119]. In contrast, the potential diagnostic value of TEP-derived RNA in canine cancer remains largely unexplored, with current research focusing on characterization of platelet RNA profiles rather than clinical validation [120]. Therefore, the use of EVs and TEPs to improve detection when ctDNA levels are low should currently be regarded as a potential, rather than established, advantage of multimarker liquid biopsy approaches. The integration of ctDNA with CTCs, EVs, TEPs, and other circulating biomarkers represents a promising future strategy that could potentially improve diagnostic and prognostic performance; however, this multi-analyte approach remains to be validated, particularly in veterinary oncology.

Despite their apparent potential, all four biomarker classes are limited by pre-analytical and analytical variability, biological heterogeneity, and incomplete standardization, particularly in veterinary oncology. For TEPs specifically, challenges include variability in platelet isolation protocols and potential confounding effects of systemic inflammatory or thrombotic conditions. Similar limitations apply to EV isolation and characterization, as well as to the low abundance and technical enrichment challenges associated with CTC detection and the low fractional abundance of ctDNA in early-stage disease [44,86,87,94,107,109,118].

At present, the main barriers to routine implementation of liquid biopsy in veterinary oncology include insufficient analytical standardization, limited validation studies, tumor-related biological heterogeneity, and reduced sensitivity in early-stage disease. Future diagnostic strategies are likely to benefit from multi-analyte approaches integrating ctDNA, CTCs, EVs, and TEPs to improve overall diagnostic accuracy, reliability, and clinical utility. Such integrative liquid biopsy frameworks have the potential to become a significant component of future precision veterinary oncology [24,87,107,121]. A comparative overview of main liquid biopsy biomarkers is presented in Table 2.

## 5. Multicancer Early Detection Tests (MCED)

MCED tests represent an emerging class of liquid biopsy-based assays designed for the detection of multiple cancer types in asymptomatic individuals. These technologies are intended to complement existing organ-specific screening strategies by enabling simultaneous assessment of a broad spectrum of malignancies from a single peripheral blood sample [126,127]. Reported performance characteristics of MCED platforms vary considerably across assays and validation studies, reflecting differences in study design, target cancer types and stages, and characteristics of the study populations [128]. Although high specificity has been reported for several MCED approaches, these estimates should be interpreted in the context of the specific assay and validation cohort rather than generalized across platforms. In population screening settings, predictive performance, particularly positive predictive value (PPV), is strongly prevalence-dependent and represents a key limitation for real-world implementation [128].

From a translational perspective, MCED testing reflects a shift toward systemic cancer detection using circulating molecular biomarkers rather than organ-specific or imaging-based approaches. Importantly, this concept is also beginning to translate into veterinary medicine, with early studies demonstrating the potential of circulating nucleic acids and extracellular vesicles as non-invasive cancer biomarkers in dogs [90], and the recently reported Cancer Lifetime Assessment Screening Study in Canines (CLASSiC) representing the first prospective study to evaluate NGS-based liquid biopsy for cancer screening in dogs [129]. However, while analytical performance has been encouraging, clinical utility in terms of mortality reduction has not yet been demonstrated in randomized controlled trials, and ongoing prospective studies continue to evaluate its impact on long-term outcomes [126,127,128].

### 5.1. DNA-Based MCED Approaches

DNA-based MCED strategies represent the most clinically advanced class of liquid biopsy assays. They primarily rely on ctDNA mutation profiling, DNA methylation signatures, and cfDNA fragmentation patterns. ctDNA consists of short cfDNA fragments carrying tumor-derived somatic alterations, including point mutations, copy number changes, and structural variants [88,90]. In early-stage disease, ctDNA is often present at extremely low fractional abundance (<0.1%), which imposes fundamental limitations on detection sensitivity [90]. Tumor shedding is highly heterogeneous and influenced by biological and anatomical factors such as vascularization, tumor burden, immune interaction, and tissue localization [128]. A major confounding factor is clonal hematopoiesis of indeterminate potential (CHIP), which introduces age-related somatic mutations into cfDNA and may reduce assay specificity in older populations [130]. DNA methylation-based approaches provide a complementary epigenetic signal for cancer detection. Aberrant methylation patterns arise early in tumorigenesis and reflect tissue-specific epigenetic remodeling. Importantly, methylation signatures enable tissue of origin (TOO) inference, although accuracy varies depending on tumor type and training datasets [92,131]. Fragmentomics-based approaches analyze cfDNA fragmentation patterns associated with changes in chromatin organization in cancer cells. Tumor-derived cfDNA exhibits altered fragment size distributions and nucleosome positioning, which can improve detection sensitivity, particularly in tumors with low ctDNA shedding [132].

### 5.2. RNA-Based MCED Approaches

RNA-based biomarkers, including microRNAs and extracellular vesicle-associated RNA, provide additional molecular information complementary to DNA-based signals. MicroRNAs are relatively stable in plasma and demonstrate tissue-associated expression patterns, supporting their potential role in cancer detection [89]. However, RNA-based MCED approaches remain largely investigational due to methodological variability, pre-analytical instability across platforms, and lack of standardization in assay development and validation [89]. At present, RNA-based strategies are considered adjunctive rather than standalone diagnostic tools within MCED frameworks.

### 5.3. Protein-Based MCED Approaches

DNA-based MCED strategies represent one of the most clinically advanced classes of liquid biopsy assays. They primarily rely on ctDNA mutation profiling, DNA methylation signatures, and cfDNA fragmentation patterns. ctDNA consists of tumor-derived cfDNA fragments carrying somatic alterations, including point mutations, copy number alterations, and structural variants [88]. In early-stage disease, ctDNA may be present at very low fractional abundance, imposing fundamental limitations on detection sensitivity [44]. Tumor shedding is highly heterogeneous and may be influenced by biological and anatomical factors, including tumor burden, vascularization, tissue of origin, and interactions with the tumor microenvironment [2]. A major confounding factor is clonal hematopoiesis of indeterminate potential (CHIP), which can introduce non-tumor-derived somatic mutations into cfDNA and complicate the interpretation of mutation-based assays, particularly in older individuals [130].

DNA methylation-based approaches provide a complementary epigenetic signal for cancer detection. Aberrant methylation patterns arise during tumorigenesis and can reflect tissue-specific epigenetic alterations. Methylation signatures can also support tissue-of-origin (TOO) inference, although performance varies across cancer types and according to the characteristics of the training and validation datasets [131]. Fragmentomics-based approaches analyze genome-wide cfDNA fragmentation patterns associated with alterations in chromatin organization in cancer. Tumor-derived cfDNA exhibits characteristic changes in fragment size distributions and nucleosome-associated fragmentation patterns, which can be exploited for cancer detection [92].

### 5.4. Integrated MCED Platforms and Clinical Evidence

Contemporary MCED strategies employ a range of genomic, epigenomic, proteomic, and fragmentation-based signals, with some platforms integrating multiple biomarker classes to improve diagnostic performance across heterogeneous tumor types.

1. CancerSEEK

CancerSEEK combines ctDNA mutation profiling with protein biomarkers to detect eight cancer types, including ovarian, liver, stomach, pancreatic, esophageal, colorectal, lung, and breast cancers [100]. In validation studies, sensitivity ranged from approximately 33% to 98%, with the highest performance in ovarian cancer and the lowest in breast cancer. Overall specificity exceeded 99% [100]. After adjustment for confounding benign and inflammatory conditions, overall sensitivity across tumor types has been estimated at approximately 55% [100,128]. Tissue-of-origin prediction was achieved in approximately 83% of cancer-positive cases.

2. Targeted error-corrected sequencing

Targeted error-corrected sequencing (TEC-Seq) enables highly sensitive detection of low-frequency ctDNA variants and has demonstrated sensitivity of approximately 59–71%, depending on cancer type and stage (including localized disease) [81]. The positivity of ctDNA has been demonstrated to be associated with a poorer clinical outcome, including a reduction in progression-free survival and OS. This finding serves to support the notion that ctDNA positivity is a significant prognostic factor [133].

3. PanSeer

PanSeer is a DNA methylation-based assay that was evaluated for cancer detection in both clinically diagnosed and pre-symptomatic individuals. In post-diagnosis cancer patients, PanSeer achieved a sensitivity of approximately 88% with a specificity of 96%. In a longitudinal cohort, the assay was additionally evaluated in 143 apparently healthy individuals who were subsequently diagnosed with cancer within four years of blood collection, achieving a sensitivity of approximately 95% in this pre-diagnosis group [134]. These findings suggest the potential of methylation-based assays for detecting cancer before conventional clinical diagnosis; however, the clinical utility of such approaches requires further prospective validation, particularly with respect to diagnostic localization and downstream clinical management [134].

4. Galleri

The Galleri test uses targeted bisulfite sequencing of more than 100,000 informative cfDNA methylation regions combined with machine learning for cancer detection and tissue-of-origin (TOO) localization [131]. In the CCGA study, stage I–III sensitivity was 67.3% for a pre-specified set of 12 cancer types, with stage-specific sensitivities of 39% for stage I, 69% for stage II, and 83% for stage III [131]. Overall sensitivity across all cancer types for stage I–III disease was 43.9%, while TOO was correctly predicted in approximately 93% of cancer-positive cases [131]. The study included more than 50 cancer types, although diagnostic performance varied according to cancer type and disease stage [131].

5. DELFI

Fragmentomics-based approaches such as DELFI analyze genome-wide cfDNA fragmentation patterns associated with chromatin accessibility changes in cancer. Reported sensitivity is approximately 73% for stage I–III cancers, with specificity of 98% and tissue-of-origin prediction accuracy of approximately 75% [92].

### 5.5. Clinical Translation, Limitations, and Veterinary Perspectives

TOO prediction is a key component of MCED interpretation, enabling targeted diagnostic work-up following a positive test result. Most approaches rely on machine learning models trained on methylation or fragmentation features, with methylation-based methods generally demonstrating higher robustness due to stronger biological specificity [92,131]. Despite encouraging analytical performance, several key limitations currently constrain clinical implementation. A fundamental biological constraint is the low abundance of ctDNA in early-stage disease, which limits sensitivity even with highly optimized sequencing approaches [44]. Tumor shedding heterogeneity further reduces detectability across cancer types and anatomical sites [2]. Although specificity is high (98–99%), even low false-positive rates may translate into a considerable number of follow-up diagnostic procedures in low-prevalence screening populations, highlighting the importance of PPV as a key performance metric [128]. Biological confounders, including clonal hematopoiesis and inflammatory conditions, further contribute to false-positive signals and reduce real-world specificity [130]. Standardized diagnostic pathways following positive MCED results are not yet established, leading to variability in imaging strategies, biopsy decisions, and clinical workflows. The existence of overdiagnosis and lead-time bias are additional limitations that must be considered. While MCED facilitates the early detection of certain malignancies, the evidence base is inconclusive regarding whether this translates consistently into enhanced overall or cancer-specific survival across tumor types. However, there is currently a paucity of randomised controlled trial evidence demonstrating a reduction in mortality [126,127,128].

In the domain of veterinary oncology, technologies pertaining to MCED remain in the early stages of translational development. The absence of established population screening programmes and standardised diagnostic pathways in veterinary oncology is a notable distinction from human medicine. The feasibility of ctDNA-based detection in canine cancers is acknowledged, but it is strongly dependent on tumor stage and biological aggressiveness. Higher detection rates are observed in advanced disease, and reduced sensitivity is noted in localized tumors. The comparative overview of MCED tests in human and veterinary oncology is presented in Table 3.

The CANDiD (CANcer Detection in Dogs) framework demonstrates stage-dependent performance of cfDNA-based detection in canine malignancies [135]. Overall detection rate was 61.9%, increasing to 85.4% in highly aggressive cancers. Localized disease showed the lowest performance (19.6%), while advanced and metastatic disease reached up to 87.5% [135].

**Figure 1:**
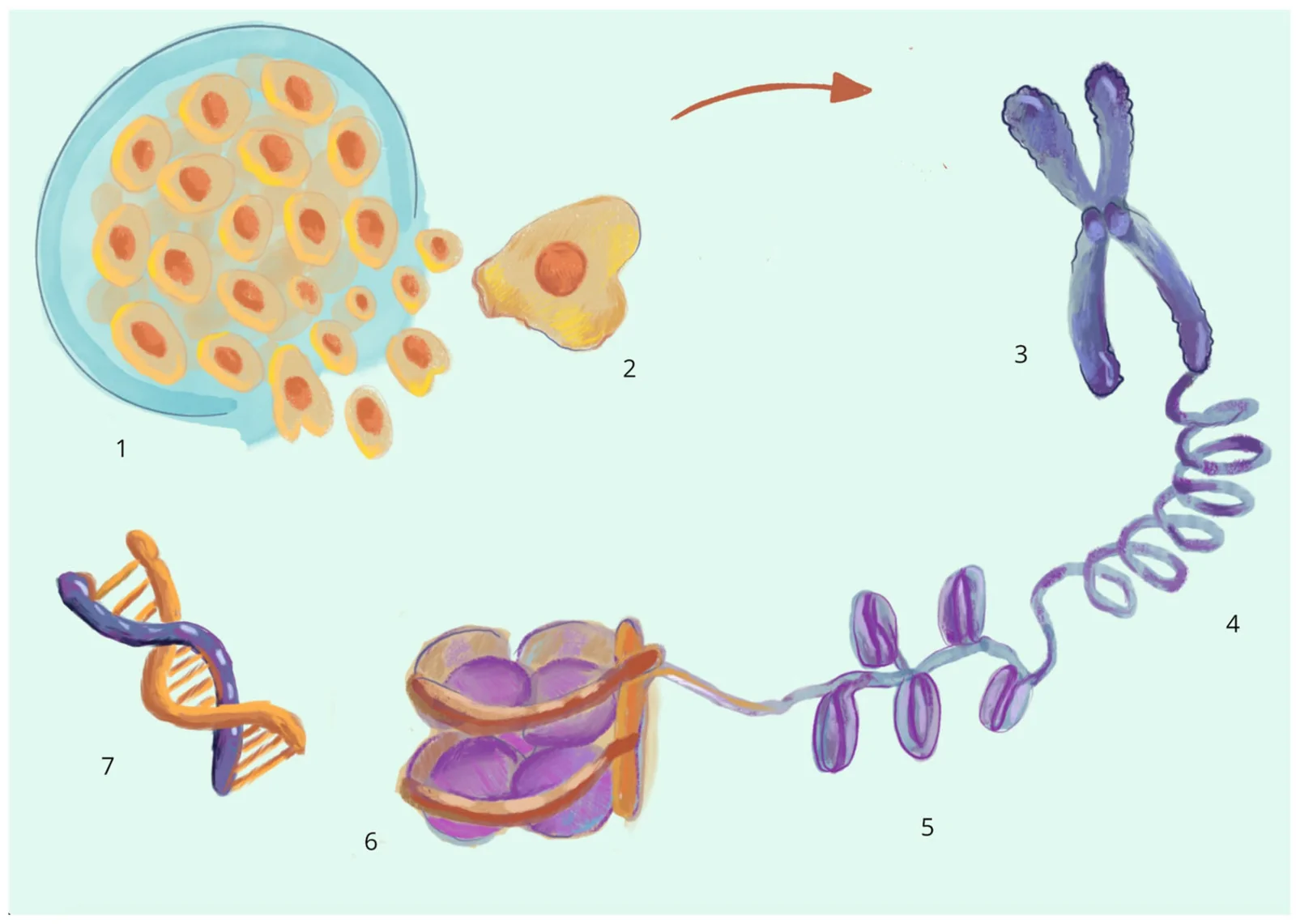
Schematic and simplified representation of chromatin organization and its fragmentation during apoptosis or necrosis. (1) Tumor tissue, (2) tumor cell, (3) chromosome, (4) chromatin looping, (5) chromatin, (6) nucleosome, and (7) cfDNA. Cell death is accompanied by chromatin fragmentation and the release of nucleosomes and cfDNA into the extracellular environment and subsequently into the circulation, where they can be detected as circulating biomarkers. Elevated levels of circulating nucleosomes and cfDNA may be observed in cancer but are not specific to malignancy and may also result from non-neoplastic cell death, inflammation, infection, tissue injury, and other pathological conditions.

In addition, nucleosome-based liquid biopsy (Figure 1), using the Nu.Q assay, has demonstrated an overall sensitivity of 49.8% and specificity of 97% for cancer detection in dogs [133]. Performance varies substantially among tumor types, with particularly low sensitivity for mast cell tumors (19.05%) and osteosarcoma (34.69%) [133]. A comparison of MCED tests is presented in Table 4.

Another commercially available MCED assay, OncoK9 by PetDx, based on ctDNA analysis, previously demonstrated promising diagnostic performance in detecting multiple canine malignancies and represented one of the first clinically available liquid biopsy screening tests for dogs. However, despite its initial commercial introduction and clinical validation, the test has been unavailable since 2024, reportedly due to company-related financial issues rather than limitations of the underlying technology. Consequently, although it is no longer accessible in routine veterinary practice, the published validation studies continue to provide valuable evidence supporting the feasibility of MCED approaches in canine oncology [136].

At present, evidence remains insufficient to determine whether MCED-based strategies improve survival or quality of life in companion animals [126,128]. Therefore, these approaches remain investigational but provide important comparative oncology insights relevant to translational cancer research [134,137].

**Table 3:** Comparative overview of MCED tests in human and veterinary oncology [88,90,128,132,134,138,139,140].

Advantages and Limitations of	Human Oncology	Veterinary Oncology
Clinical application	Advantages: Detects multiple cancer types in asymptomatic individuals, complements organ-specific screening. Limitations: PPV depends on prevalence, risk of overdiagnosis, false positives may lead to unnecessary follow-up procedures.	Advantages: MCED approaches may offer opportunities for earlier detection of aggressive cancers and could support translational and comparative oncology research. Limitations: No population screening programs, unknown impact on survival or quality of life, limited data.
Biomarker types	Advantages: Multi-biomarker integration (DNA, RNA, protein) allows complementary signals. Limitations: RNA biomarkers mainly adjunctive; protein biomarkers alone have lower specificity.	Advantages: DNA-based detection feasible; nucleosome assays show promise. Limitations: RNA/protein biomarkers largely experimental; standalone protein tests have lower specificity.
Example platforms	Advantages: CancerSEEK, PanSeer, Galleri, and DELFI have undergone varying degrees of analytical and/or clinical evaluation. Some have been assessed in prospective or clinical studies, although the extent of validation and clinical maturity differs substantially among platforms. Limitations: Sensitivity varies by cancer type/stage, TOO prediction depends on large datasets, false positives in low-prevalence populations.	Advantages: Potential future application for early detection and molecular characterization of aggressive cancers (CANDiD, Nu.Q) Limitations: Lower sensitivity in early/localized tumors, limited TOO prediction, small reference datasets.
Sensitivity and Specificity	Advantages: Selected MCED studies have reported high specificity, approaching 98–99%, although performance varies by platform and study population. Limitations: Limited sensitivity in early-stage cancers, PPV depends on prevalence.	Advantages: Reasonable detection in advanced/aggressive tumors. Limitations: Low sensitivity in early/localized cancers, species heterogeneity, limited reference data.
TOO prediction	Advantages: ML-based models have demonstrated promising performance for TOO classification in selected studies. Limitations: Accuracy varies by tumor type, requires large datasets.	Advantages: Species-specific TOO prediction is a potential future application that could support diagnostic classification if adequately validated. Limitations: Currently unreliable due to limited training data and species-specific methylation patterns.
Biological factors	Advantages: Can detect low-abundance ctDNA, captures tumor heterogeneity. Limitations: Clonal hematopoiesis and inflammatory conditions can generate false positives.	Advantages: MCED approaches may provide comparative oncology insights and enable investigation of tumor-associated circulating biomarkers. Limitations: ctDNA levels low in early disease, heterogeneity requires tumor-specific panels.
Technical and methodological factors	Advantages: Selected human MCED platforms have developed increasingly standardized workflows, scalable assay formats, and relatively large reference or validation cohorts. Limitations: High cost; follow-up procedures required for positives. Standardization remains an important challenge across the field, including differences in sample processing, assay design, bioinformatic pipelines, reference populations, and validation criteria.	Advantages: cfDNA detection feasible, useful for translational research. Limitations: Lack of standardized workflows, pre-analytical variability, need for species-specific validation.

**Table 4:** Comparison of Selected MCED Tests [92,100,135,139,141].

MCED Test	Method	Cancer Types Analyzed	Detection of TOO	Reference
CancerSEEK	detection of ctDNA	8	83% of cases	[100]
PanSeer	detection of ctDNA methylation	5	not able	[134]
Galleri test	detection of DNA methylation	50	93% of cases	[139]
DELFI test	cfDNA fragmentation	7	75% of cases	[92]
CANDiD *	detection of ctDNA	8	Not specified	[135]
Nu.Q *	elevated levels of nucleosomes	7	not able	[141]

The tests designed for cancer detection in dogs are marked with *.

## 6. Discussion

Despite the fact that tissue biopsy remains the gold standard for cancer diagnosis, its clinical utility may be limited by the invasive nature of the procedure, difficulties associated with tumor localization, and reduced applicability in early-stage disease detection [4,142]. In this context, liquid biopsy has emerged as a promising complementary diagnostic approach with potential applications in cancer screening, disease monitoring, and therapeutic management [5].

The clinical value of liquid biopsy-based MCED tests is contingent on their capacity for early-stage cancer detection, high specificity, and accurate TOO identification [131,143]. However, reported sensitivities are generally lower for early-stage malignancies [93]. This may result in false reassurance following negative test results and potentially delay further diagnostic evaluation or screening procedures [99]. Conversely, false-positive findings may lead to additional diagnostic investigations and may contribute to patient anxiety and overdiagnosis [99,128]. It is important to note that elevated circulating biomarkers, such as cfDNA, are not exclusively associated with malignancy. Indeed, they may also occur in inflammatory conditions or after physical exercise [93,135].

Despite these limitations, liquid biopsy is considered a potentially valuable tool for monitoring disease progression and treatment response [30]. Associations between circulating biomarker levels and survival outcomes have also been reported, supporting their potential prognostic relevance; however, such associations should not be interpreted as evidence that liquid biopsy-guided therapeutic decision-making improves treatment outcomes or survival, particularly in veterinary patients [1]. Consequently, liquid biopsy may become an important component of personalized patient management, including early cancer detection and longitudinal disease monitoring, but its impact on clinical outcomes remains to be established. Future studies investigating additional biomarkers, including methylation patterns and exosome analysis, may further improve the diagnostic and prognostic performance of these approaches [4].

## 7. Future Perspectives

In veterinary medicine, liquid biopsy, particularly ctDNA-based analysis, represents a promising complementary approach for cancer detection and disease monitoring, with potential applications in earlier diagnosis and treatment assessment. However, clinical implementation remains limited by insufficient sensitivity in early-stage and low-burden disease, variability in ctDNA shedding among tumor types, difficulties in accurate identification of the tissue of origin (TOO), and the lack of standardized analytical and clinical workflows. Therefore, liquid biopsy should currently be considered an emerging and primarily investigational technology that complements, rather than replaces, tissue-based diagnosis and conventional imaging.

Future progress in this field will likely depend on prospective clinical validation, assay standardization, and the integration of complementary circulating biomarkers. Multi-analyte approaches incorporating ctDNA, CTCs, EVs, TEPs, and epigenetic signatures such as DNA methylation patterns may provide a broader representation of tumor biology and potentially improve diagnostic performance. Advances in NGS and PCR technologies may further facilitate the development of sensitive and reproducible assays, although their clinical utility will need to be established through appropriately designed validation studies.

In the domain of veterinary medicine, liquid biopsy presents a potentially valuable opportunity to address the limited availability of structured cancer screening strategies, particularly in companion animals such as dogs. In contrast to human oncology, where organized screening programs enable the detection of malignancies in asymptomatic individuals, veterinary oncology lacks standardized screening strategies for the vast majority of neoplastic diseases. Consequently, cancer in companion animals is frequently diagnosed incidentally during routine examinations or after the onset of non-specific clinical signs, such as weight loss, lethargy, or decreased appetite, when the disease may already be advanced [135,144,145]. This disparity has stimulated interest in translational diagnostic technologies that could facilitate earlier cancer detection while also contributing to comparative oncology research [146,147].

Although tissue sampling remains the gold standard for definitive cancer diagnosis, its invasiveness and limited feasibility for certain tumor locations highlight the need for complementary diagnostic approaches. Diagnostic imaging remains central to tumor detection, staging, and treatment planning in veterinary medicine, although its sensitivity may be insufficient for early-stage lesions or minimal residual disease [18,148]. Some imaging procedures also require sedation or general anesthesia, which may limit their use in certain clinical settings. Liquid biopsy may therefore offer a minimally invasive complementary source of molecular information, but its added clinical value relative to established diagnostic approaches requires further investigation.

Among emerging molecular approaches, NGS has demonstrated considerable potential for the molecular characterization of canine and feline tumors, particularly within comparative oncology [1,55,60]. However, routine clinical implementation remains constrained by cost, bioinformatic requirements, inter-platform variability, and the need for standardized analytical workflows [21,31,42]. Spectroscopic techniques have likewise shown promise as minimally invasive complementary diagnostic tools, but broader clinical adoption remains limited by methodological variability, challenges in data interpretation, and the need for large-scale validation [1,34,35,37,60].

Economic considerations should also be incorporated into future clinical development. Although liquid biopsy may ultimately prove cost-effective if it facilitates earlier diagnosis or reduces the need for invasive procedures, the current expense of molecular testing may limit accessibility in routine veterinary practice, particularly where diagnostic costs are borne entirely by animal owners. Future studies should therefore assess not only analytical performance but also clinical utility, cost-effectiveness, feasibility, and impact on patient management.

Ultimately, successful translation of liquid biopsy into veterinary oncology will require rigorous analytical and clinical validation, harmonization of pre-analytical and analytical procedures, and appropriately powered prospective studies. Particular attention should be given to defining the performance of individual biomarkers across tumor types and disease stages, establishing clinically meaningful thresholds, and determining whether biomarker-guided decisions provide measurable benefits beyond conventional diagnostic and monitoring strategies. Such evidence will be essential to determine whether liquid biopsy can progress from an exploratory research tool to a clinically useful component of veterinary oncology.

## 8. Conclusions

Liquid biopsy represents a promising, minimally invasive approach with potential applications in cancer detection and disease monitoring in veterinary medicine. Current evidence, however, remains limited, and most applications should still be regarded as investigational rather than clinically established. Further prospective validation, methodological standardization, and evaluation of clinical utility are required to determine the role of liquid biopsy in routine veterinary oncology. Future advances in multi-analyte strategies may enhance their diagnostic potential, but whether these approaches translate into improved treatment decisions or patient outcomes remains to be established.

## Figures and Tables

**Table 1:** Comparative framework of diagnostic development [1,21,22,25,30,60].

Domain	Human Oncology	Veterinary Oncology	Key Limitation in Veterinary Context
Screening programs	Established (selected cancers)	Rare/non-standardized	Lack of population-based screening
Imaging	Widely standardized	Widely used but late-stage detection	Late presentation bias
Tissue biopsy	Gold standard	Gold standard	Invasiveness, feasibility in repeated sampling
Molecular diagnostics	Clinically integrated	Emerging/limited availability	Cost and access barriers
Liquid biopsy	Increasing clinical adoption	Mostly research-based	Lack of validation and standardization

**Table 2:** Comparative overview of main liquid biopsy biomarkers.

Feature	ctDNA	CTCs	EVs	TEPs	References
Biological origin	Fragmented DNA released from apoptotic/necrotic tumor cells	Intact viable tumor cells released from primary or metastatic lesions	Membrane-bound vesicles secreted by tumor and normal cells	Platelets “educated” by tumor through uptake of RNA, proteins, EVs and signaling molecules	ctDNA: [22,43,44]; CTCs: [118,122]; EVs: [107,109]; TEPs: [86,111,123]
Nature of analyte	Cell-free nucleic acid	Whole cells	Nucleic acids, proteins, lipids, metabolites enclosed in vesicles	Platelet RNA profile, proteins, spliced transcripts	ctDNA: [22,43]; CTCs: [118,122]; EVs: [107,109]; TEPs: [86,111]
Type of biological information	Genetic and epigenetic alterations (mutations, methylation patterns)	Cellular phenotype, morphology, protein expression, viability	Intercellular communication signals and tumor-derived molecular cargo	Tumor-induced transcriptomic and splicing signatures reflecting tumor activity	ctDNA: [22,30,43]; CTCs: [118,122]; EVs: [107,109]; TEPs: [86,111,123]
Analytical performance/detectability	Assay-dependent, can be high with sensitive molecular methods, but varies with tumor type, tumor burden, ctDNA fraction, and assay design	Detection is challenging because CTCs are rare; analytical performance depends strongly on enrichment and detection methodology	Variable and strongly dependent on isolation, characterization, and downstream analytical methods	Potentially strong performance in RNA-based assays, but dependent on platelet isolation, RNA quality, assay design, and analytical validation	ctDNA: [22,30,44]; CTCs: [118,122]; EVs: [107,109]; TEPs: [86,111,123]
Suitability for early-stage disease	Limited sensitivity in low tumor burden settings and varies substantially among tumor types	Can be present in early disease, but their low abundance makes reliable detection challenging; stage-specific clinical validation remains limited	Potential for early detection based on tumor-associated molecular cargo, but stage-specific clinical sensitivity remains insufficiently validated	Promising investigational potential for early detection, clinical validation remains limited	ctDNA: [44,80]; CTCs: [118,122]; EVs: [9,107,109]; TEPs: [56,123]
Main technical challenges	Pre-analytical variability, low fraction in circulating free DNA (cfDNA) pool, need for high-sensitivity assays	Low abundance, complex enrichment, loss during isolation	Lack of standardization, isolation heterogeneity, contamination with non-tumor EVs	Standardization of isolation, RNA sequencing pipelines, influence of pre-analytical factors	ctDNA: [30,94]; CTCs: [118,122]; EVs: [107,124]; TEPs: [86,123]
Biological limitations	Variable shedding, influenced by tumor type and microenvironment	High inter- and intra-tumor heterogeneity, EMT-related detection loss	Heterogeneous populations, mixed cellular origin	Influence of inflammation, thrombosis, and systemic diseases	ctDNA: [22,44]; CTCs: [118,122,125]; EVs: [107,109]; TEPs: [86,123]
Standardization level	Moderate to high in human oncology; limited in veterinary medicine	Limited; no universally standardized protocols in veterinary oncology	Low, major methodological variability across studies	Currently limited, especially in veterinary oncology	ctDNA: [1,30,87]; CTCs: [118,122]; EVs: [107,124]; TEPs: [86,123]
Clinical maturity (veterinary oncology)	Emerging and comparatively advanced among liquid biopsy approaches, but veterinary clinical validation remains limited	Experimental, veterinary studies demonstrate feasibility, but clinical standardization and validation remain limited	Early exploratory stage, promising veterinary applications but substantial methodological and validation challenges remain	Investigational, promising evidence mainly from human oncology, with limited veterinary validation	ctDNA: [1,9,22,30]; CTCs: [1,118]; EVs: [107]; TEPs: [86,111,123]
Main clinical applications	Disease monitoring, MRD, therapy response, prognostication	Metastatic risk assessment, prognostic stratification (research setting)	Exploratory biomarker for diagnosis, prognosis, and tumor biology	Early detection, tumor classification, monitoring, therapeutic response	ctDNA: [1,22,24,30,88]; CTCs: [118,122]; EVs: [107,109]; TEPs: [86,123]
Key advantage	High molecular specificity and longitudinal monitoring capability	Direct cellular characterization and functional insights	Reflects tumor–microenvironment communication and stable molecular cargo	Highly stable RNA cargo, abundant and easily accessible blood component	ctDNA: [22,24,88]; CTCs: [118,122]; EVs: [107,109]; TEPs: [86,111,123]
Key limitation	Reduced sensitivity in early disease and biological variability of shedding	Extreme rarity and technical difficulty of isolation	Lack of robust clinical validation and standardization	Lack of standardization and influence of non-cancer conditions	ctDNA: [22,44,80]; CTCs: [118,122]; EVs: [107,124]; TEPs: [86,123]

## Data Availability

No new data were created or analyzed in this study. Data sharing is not applicable to this article.

## Abbreviations

The following abbreviations are used in this manuscript:

cfDNA	Circulating free DNA
cfRNA	Circulating free RNA
CHIP	Clonal hematopoiesis of indeterminate potential
CT	Computed tomography
CTCs	Circulating tumor cells
ctDNA	Circulating tumor DNA
dPCR	Digital polymerase chain reaction
EVs	Extracellular vesicles
FDA	US Food and Drug Administration
FISH	Fluorescence in situ hybridization
IHC	Immunohistochemistry
LDCT	Low-dose computed tomography
MCED	Multicancer early detection
MRD	Minimal residual disease
MRI	Magnetic resonance imaging
NGS	Next-generation sequencing
PCR	Polymerase chain reaction
PET	Positron emission tomography
PPV	Positive predictive value
PSA	Prostate-specific antigen
qPCR	Quantitative polymerase chain reaction
RT-PCR	Reverse transcription polymerase chain reaction
TEC-Seq	Targeted error-corrected sequencing
TEPs	Tumor-educated platelets
TOO	Tissue of origin
